# Mexican Microalgae Biodiversity and State-Of-The-Art Extraction Strategies to Meet Sustainable Circular Economy Challenges: High-Value Compounds and Their Applied Perspectives

**DOI:** 10.3390/md17030174

**Published:** 2019-03-18

**Authors:** Juan Eduardo Sosa-Hernández, Kenya D. Romero-Castillo, Lizeth Parra-Arroyo, Mauricio A. Aguilar-Aguila-Isaías, Isaac E. García-Reyes, Ishtiaq Ahmed, Roberto Parra-Saldivar, Muhammad Bilal, Hafiz M. N. Iqbal

**Affiliations:** 1 Tecnologico de Monterrey, School of Engineering and Sciences, Campus Monterrey, Ave. Eugenio Garza Sada 2501, CP 64849, Monterrey, N.L., Mexico; eduardo.sosa@tec.mx (J.E.S.-H.); a00823430@itesm.mx (K.D.R.-C.); a01036078@itesm.mx (L.P.-A.); a00816656@itesm.mx (M.A.A.-A.-I.); a00824289@itesm.mx (I.E.G.-R.); r.parra@tec.mx (R.P.-S.); 2 School of Medical Science, Menzies Health Institute Queensland, Griffith University (Gold Coast campus), Parklands Drive, Southport, QLD 4222, Australia; ishtiaq.ahmed@griffithuni.edu.au; 3 School of Life Science and Food Engineering, Huaiyin Institute of Technology, Huaian 223003, China; bilaluaf@hotmail.com

**Keywords:** microalgae, biodiversity, bioactive compounds, green extractions, pharmaceutical, secondary metabolites, biofuels

## Abstract

In recent years, the demand for naturally derived products has hiked with enormous pressure to propose or develop state-of-the-art strategies to meet sustainable circular economy challenges. Microalgae possess the flexibility to produce a variety of high-value products of industrial interests. From pigments such as phycobilins or lutein to phycotoxins and several polyunsaturated fatty acids (PUFAs), microalgae have the potential to become the primary producers for the pharmaceutical, food, and agronomical industries. Also, microalgae require minimal resources to grow due to their autotrophic nature or by consuming waste matter, while allowing for the extraction of several valuable side products such as hydrogen gas and biodiesel in a single process, following a biorefinery agenda. From a Mexican microalgae biodiversity perspective, more than 70 different local species have been characterized and isolated, whereas, only a minimal amount has been explored to produce commercially valuable products, thus ignoring their potential as a locally available resource. In this paper, we discuss the microalgae diversity present in Mexico with their current applications and potential, while expanding on their future applications in bioengineering along with other industrial sectors. In conclusion, the use of available microalgae to produce biochemically revenuable products currently represents an untapped potential that could lead to the solution of several problems through green technologies. As such, if the social, industrial and research communities collaborate to strive towards a greener economy by preserving the existing biodiversity and optimizing the use of the currently available resources, the enrichment of our society and the solution to several environmental problems could be attained.

## 1. Introduction

**Figure 1:**
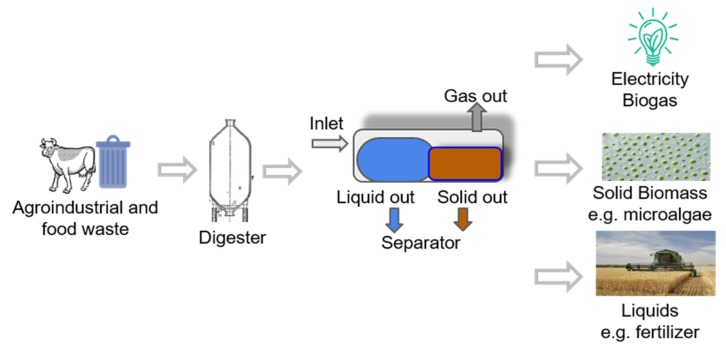
Microalgae biotechnology valorization scheme to produce energy and bio-compounds from agro-industrial and food waste.

Current needs demand high-level bio-compounds production coped with cutting-edge biotechnology. Several strategies to produce valuable compounds addressed by pharmaceutical and food industry rely on microorganism production. However, other bioactive products still rely on synthetic production processes. Plants, yeasts, bacteria, fungi, and microalgae are the most used organisms to produce such compounds naturally. Microalgae have a large number of species, and little is known about their potential uses in comparison to the diversity that is reported every day [1]. Microalgae are one of the most used bio-systems to produce different compounds in biotechnology (Figure 1). The utilization of microorganisms’ machinery helps to generate high-value bio-products [2]. As this is a bioprocess, it has several advantages over other techniques since it offers new environmental-friendly opportunities. The objective of this work is to compile a list of strains, show the relevance of new extraction techniques, and characterize current applications and potential future biotechnological microalgae opportunities.

Microalgae or cyanobacteria are unicellular, cenobial, pluricellular, or colonial organisms adapted to live in water systems, soils, or as symbionts [1]. Depending on the species, they live in complex systems or as individual cells and interact with light through photosynthesis producing oxygen and consuming carbon dioxide [3]. Microalgae can produce biomass containing high-value bio-compounds and at the same time bio-fixate ions, both important factors to propel microalgae biotechnological applications in the new era of environment remediation [4,5,6]. Cultivation technologies to produce biomass include open ponds, photo-bioreactors, and fermentation reactors. A lack of attention to microalgae species is evident since only a few hundred have been investigated. It is believed that at least tens of thousands exist in the world [7]. Nonetheless, some countries had already looked for food production through microalgal cultivation. Mexican Aztecs used to cultivate and consume Spirulina from Texcoco salted lake [8]. Japan has been leading since the early 1950s [9] with the first industrial-scale production of *Chlorella* for human consumption to ingest as a nutritional supplement. Leading to use *Chlorella* as the main microalgae source of dietary supplement nowadays. Harvesting and drying of its biomass use expensive centrifuges and cells need to be broken therefor 5000 Mt of *Chlorella* biomass were sold for approximately $20,000/100 kg. Other successful microalgae with great industrial production are *Spirulina*. They have several advantages over *Chlorella* since they require a little inoculum, and is not easy to get contaminated, they grow in temperatures between 15 and 38° Celsius, high pH, and alkalinity. *Spirulina* market value, plant gate, is about $10,000 per ton and its main use is for food supplement [10]. Nowadays, *Chlorella* and *Spirulina* are the principal microalgae used for nutritional supplements, and their producers value them in the global market about $40–50 per kg [11]. The price and volume relations are even higher when pure fine-chemicals are obtained from algal cultivation. β-carotene is a pigment strongly used in the United States. In the market, its price is estimated from $300 to $3,000 USD/kg, the price depends entirely on the production, the fickle market, and the product purity [12]. The animal feeding price is about $10 USD/kg in the nutraceutical sector. On the other hand, for example, the same product for human consumption is sold at $120 US/kg [13].

Microalgal biomass is also used to get biofuels. In comparison with petroleum, biomass is more expensive. Assuming the lipid dry weight content within microalgae, 29.6% (lipid/biomass), the algal biomass must be produced at the cost of US $ 152.00 per ton to be competitive with petroleum [14]. Also, in this area, microalga biomass price along with its valued compound depend on where the product is located and the market status [15]. As discussed previously, it is evident that biotechnology focused on microalgae has a substantial potential application in the industry. This review presents several Mexican microalgae strains along with novel green extraction technologies applied to extract microalgae-based high-value compounds. Then, a comprehensive list of compounds is presented within five fields of applications, to be followed by potential applications and opportunities for improvement. Finally, concluding remarks and future perspectives are summarized.

## 2. Mexican Microalgae Biodiversity

Mexico is one of the countries with the highest biodiversity in the world thanks to its geography and size that covers several latitudes. The Mesoamerica region provides very different environmental conditions to support life [16]. Algal strains from all around the country were isolated and studied by research groups across México. To our knowledge, this is the first work to gather information from microalgae found in several locations, systems, and types of waterbodies in the country that also addresses its applications and prospect its potential applications in contrast to just freshwater biodiversity [17,18]. 

The Pacific Ocean is a vast water source with the unique condition of being warm. From this Mexican litoral, in Baja California, 21 species were isolated, but *Aphanocapsa marina*, *Komvophoron* sp., and *Phormidium* sp. were selected thanks to their capacity to produce fatty acids. The remaining strains were also characterized but were not selected for aquaculture farm food [19]. Raw microalgae biomass is used as a nutraceutical product in aquaculture activities directly in the country by increasing its productivity [20]. However, Rodríguez-Palacio et al. focused on a large microalgae diversity that causes algal blooms with toxic consequences for aquatic fauna in twelve locations of the country, where the harmful toxic microalgae affect fishes. In addition, they proposed the culture of those microalgae in order to evaluate changes in water pollution [21]. A list of microalgae found in Mexican water sources and isolated is presented in Table 1. Lately, the discovery of novel microorganisms is increasing, and it is expected to continue thanks to new research groups across the country. The panorama presented by the list of microalgae suggests potential applications since they were found in various environment growth conditions including volcano ponds, salted lakes, freshwaters, and seawaters. Even in extreme physiological conditions, microorganisms are capable of producing compounds with high value. To get an advantage, biotechnological processes like production, extraction, and purification require novel and environmentally friendly methods. The next section focuses on the description of green extraction methodologies.

## 3. State-Of-The-Art of Extraction Methods

Currently, most common extraction techniques consist of non-green processes. Millions of liters of organic solvents are used in the extraction process. Consequently, interest in green extraction techniques has recently increased as they are less expensive but cope with the global tendency of green legislation [38]. These processes are named, green extraction, since they do not negatively affect the environment and take advantage of the compound properties such as polarizability, charge, structure, as well as size [38]. Such processes include microwave extraction, supercritical fluid extraction, as well as ultrasound extraction. Using the appropriated method, bioactive compounds are extracted from microalgae, and valuable molecules remain functional after an efficient extraction. A list of compounds extracted from microalgae using ultrasound, microwave, and supercritical fluid extraction methods are presented in Table 2.

### 3.1. Microwave-Based Extraction

**Figure 2:**
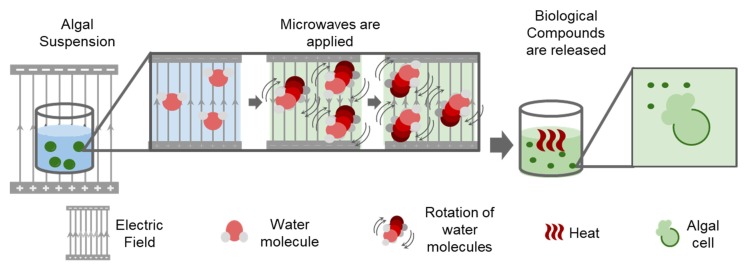
Scheme of microwave algal extraction.

Microwave extraction involves heating of samples in a polar solution such as ionic liquids, ethanol, methanol, chloroform, acetone, and ultra-pure water by placing them in an alternating electric field. The solvent molecules align according to the applied electric field and quickly increase the temperature of the samples as a result of the inter- and intramolecular friction caused by this movement. The algal cell walls break with this sudden increase in temperature and release the compounds of interest (Figure 2). The resulting extract must undergo further purification as this process does not result in the isolated compound of interest [56]. This method differs from conventional heating extraction methods as it does not depend on the diffusion rate of the mixture mass. Therefore, microwaves heat the solvent which interacts with all the sample and prevents the formation of hot-spots and allowing for more homogeneous thermoregulation of the cell mixture. Further, it is quick and has been demonstrated to be at least ten times faster than conventional bath heating extraction methods, as mentioned by Balasubramanian et al. [56]. Additionally, the same study showed that the use of this method is especially beneficial when extracting lipids from microalgae, as it allows for a final product of better quality, with higher unsaturated lipids and antioxidant concentrations in comparison to the conventional Soxhlet extraction. In a second study involving pigment extraction from *Dunaliella tertiolecta* and Cylindrotheca closterium, it was demonstrated that microwave extraction techniques are more efficient than solvent-based techniques in overcoming mechanical resistance factors that limit solvent penetration into the cells [57].

The prospects for industrial implementation of Microwave extraction techniques have been previously discussed by Vinatoru et al. [58], where advantages such as shorter loading and downloading times as well as easier maintenance of the equipment are added to the lower energy consumption and faster extraction times. However, specific advantages depend on the type of material to be extracted (as previously discussed, microwave extraction methods are more efficient for certain mixtures due to their intrinsic properties, e.g., mechanical resistance). Nevertheless, pretreatment methods such as enzymatic digestion or milling could allow for a standardized development of this extraction method by improving its efficiency for a wide range of mixtures [58]. Also, the coupling of this method with other extraction techniques could also improve its efficiency. Still, the feasibility of scaling up microwave extraction techniques into an industrial level is still under research; because its high energy operating costs might present a major drawback of this process. For instance, the use of enzymes is expensive, especially for large scale applications when competing against other methods [59]. Moreover, this method cannot complete the separation process by itself and usually requires a subsequent centrifugation or filtration process [60]. 

### 3.2. Supercritical Fluid-Based Extraction

**Figure 3:**
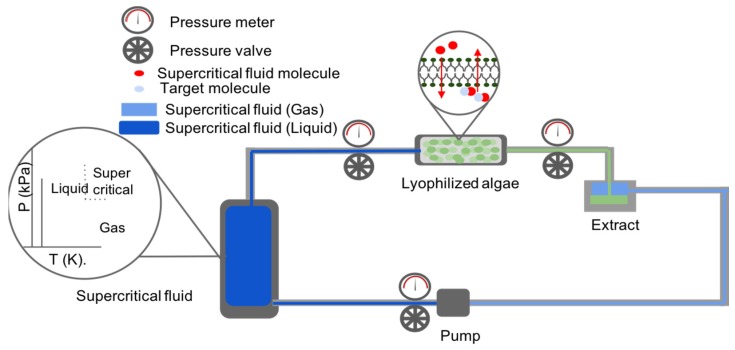
Scheme of the supercritical fluid extraction process in a closed system.

This extraction method depends on the usage of supercritical fluids (e.g., carbon dioxide, water, methane, ethane, methanol, ethanol, acetone, and nitrous oxide), induced either by temperature or pressure excitation, for the recovery of valuable products. Such fluids are capable of crossing the cellular membrane and wall of microalgae and solubilize internal metabolites to extract them from the cell (Figure 3). In most cases, supercritical fluids’ induction conditions range between $P_{C}<P\leq 6P_{C}$ for pressure and $1.01T_{C}<T\leq 1.4T_{C}$ for temperature in order to allow for an energy efficient extraction (where, $P_{C}$ is the critical pressure constant and $T_{C}$ is the critical temperature constant for the supercritical fluid) [61]. The most common and preferred solvent used for this method is CO_2_ as it has an ambient critical temperature, is non-flammable, is chemically inert, non-toxic, and inexpensive [61]. As such, it is able to meet the ecological and chemical safety standards required for the extraction of high purity biological products (such as pigments for cosmetics and antiviral agent in medicine) as it leaves no harmful solvent residues and prevents thermal degradation of sensitive products. Moreover, with the addition of a polar entrainer (such as water) the solvent is able to dissolve polar compounds as well [61], thus allowing for a robust extraction procedure. 

The main advantage this method presents when compared to the conventional Soxhlet extraction method is the fact that it prevents the deposition of residual toxic matter in the extract, while also proving to be quite economical. The mentioned characteristics are because CO_2_ can reach a supercritical condition at a temperature of 31 °C (conventionally a temperature of 35 °C is preferred for extraction of biological materials). This critical temperature allows the liquid CO_2_ to be used without the consumption of excessive amounts of energy, and with a minimal reduction in temperature, most of the solvent will easily precipitate. Further, it is easily affordable, having a nominal price of 2.65 USD/kg in the year 2017 [62]. Also, as explained before, the non-extreme range of temperatures in which this method can operate prevents the degradation of valuable biological products, thus allowing for a high yield.

A recent study also analyzed the possibility of combining the use of supercritical fluid extraction with cold pressing to improve the extraction of fennel by supercritical fluid extraction alone [63]. The combination of these two extraction methods was originally developed by Johner et al. [64] for the extraction of pequi. This researchers demonstrated some promising results: a faster extraction rate with the consumption of less solvent. These results were validated with the fennel extraction, where the overall yield extraction was improved by 24.5%. Hatami et al. [63] inferred that this might be due to the increased exposure of oil to supercritical CO_2_ caused by the release of the substance from the compressed matter. These results also demonstrate that supercritical extraction can reduce operational time and costs while increasing yields if combined with other extraction procedures such as those including solvents like acetone or ethanol. All these factors proved that supercritical fluid extraction might be a promising alternative for a greener future. Nevertheless, supercritical fluid extraction machinery usually represents a high capital cost, regardless of the economic viability in the long run. For example, according to De Aguilar [62], Supercritical Fluid Extraction (SFE) units’ nominal prices ranged between 530,000 to 2,600,000 USD in 2017. Further, while temperature extraction conditions are amiable, pressure operating parameters still need to be decreased for general industrial implementation. While previous studies have been able to optimize industrial relevant conditions for supercritical extraction of lipids from *Scenedesmus obliquus, Chlorella protothecoides and Nannochloropsis salina* [65], optimal pressure conditions can range between 30 MPa and 50 MPa in some cases [59], thus representing an area of opportunity for further optimization of the standardized application. Polyunsaturated fatty acids like ω-3 and ω-6 types have been successfully extracted by SFE using CO_2_ and n-butane. Feller et al. [66] found a significant relationship between the content of carotenoids and the respective antioxidant activity. They used *Phaeodactylum tricornutum*, *Nannochloropsis oculata*, and *Porphyridium cruentum* strains and attributed the antioxidant activity of the marine microalgae to the carotenoid compounds [66]. In the case of subcritical n-butane, the procedure is the same as for the supercritical system. The difference relays in the control conditions using n-butane at 15 bar, 40 °C and solvent flow rate of 3 mL min^−1^ [66].

### 3.3. Ultrasound-Based Extraction

**Figure 4:**
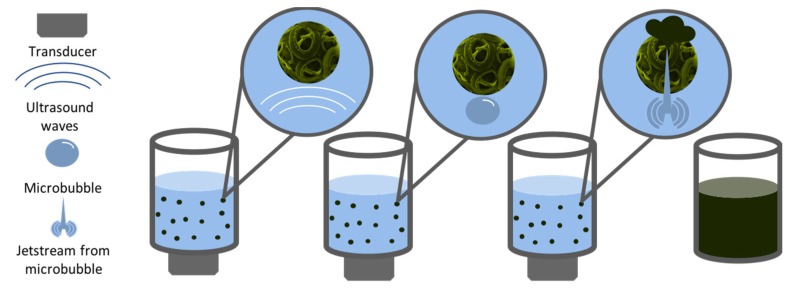
Schematic representation of the steps involved in the ultrasound-assisted extraction method.

**Figure 5:**
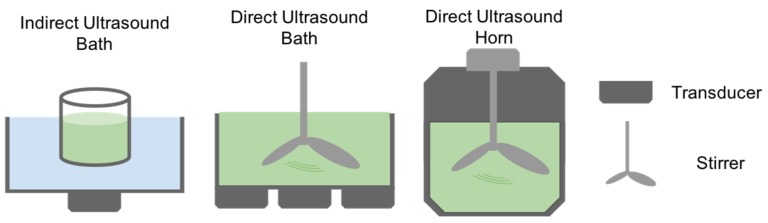
Different methods involved in ultrasound assisted extraction.

The technique of Ultrasound Extraction uses high-frequency sound waves to disrupt algal cell walls leading to the subsequent release of the compound of interest. As shown in Figure 4, the process depends on a physical phenomenon called cavitation where the disruption of the solvent caused by the sound waves, creates small bubbles. The bubbles generate strong jet streams as they implode by the effect of the acoustic cavitation force. When the bubble is close to a cell, allow for the puncturing of the cell wall and membrane [67]. The particular solvent used for this extraction method depends on the physical characteristics of the target compound, and subsequent fractionations are needed to isolate the compound of interest. Ultrasound extraction may occur directly as well as indirectly. Indirect ultrasound consists of placing a transducer touching the outer surface of a water bath. In the case of direct ultrasound extraction, the transducer can be close to the container where the bath occurs or in the form of an ultrasound horn in the sample (Figure 5). The advantages of this method include faster and substantial yields than other conventional techniques and moderate to low costs, in addition to minimal toxicity [68]. According to Kledjus et al. [69], it also allows for a more efficient extraction in freshwater algae species. On the other hand, the technique might negatively affect the quality of the oils as well as the integrity of polyunsaturated fatty acid rich oils. Furthermore, it cannot be scaled up [67].

## 4. Current Applications 

**Figure 6:**
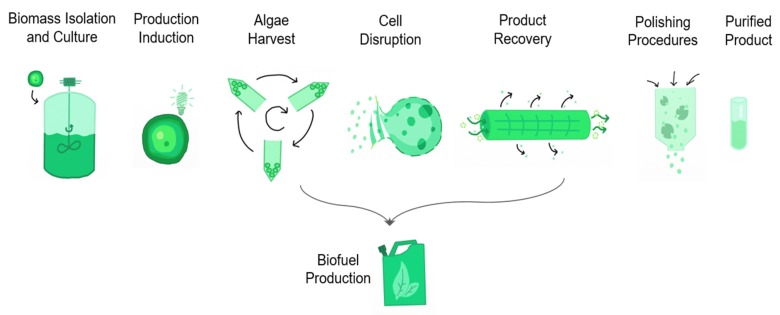
Scheme of the biotechnological process to produce bio-compounds from microalgae biomass.

Cyanobacterial have already shown potential applications in biotechnology, biomedicine, food, biofuel, fertilizers, pigments, waste treatment, among others. The production (general process scheme is presented in Figure 6) of various secondary metabolites includes toxins, antioxidants, vitamins, bio-adsorbents, enzymes, and pharmaceuticals. Considering cyanobacterial pluripotential biotechnological uses, an overview is presented. First, a list of microalgae strains found in Mexico and the bioactive compounds produced are presented in Table 3, followed by the description of the current applications of relevant works with the mentioned microalgae.

### 4.1. Pigments - Phycobilins, Lutein, and Carotenoids

Phycobilins are produced only by algae such as *Spirulina* [114]. Phycobilins are photosynthetic pigments bonded to water-soluble proteins, building the so-called phycobiliproteins. Phycocyanin, phycoerythrin, and allophycocyanin are water-soluble and have a wide range of applications including food and cosmetic colors, as fluorescent tags for use in flow cytometry and immunology [52]. Other possible applications are as antioxidants in cosmetics, a component of functional foods, and photosensitizers in photodynamic cancer therapy [52,115]. Phycobilins are found in the stroma of chloroplasts of cyanobacteria, rhodophytes (red algae), glaucophytes, and some cryptomonads [116]. They forward the energy of the harvested light to chlorophylls for photosynthesis. Similar to carotenoids, those proteins serve as “secondary light-harvesting pigments” [117]. Besides these highly sophisticated applications as chemical tags, phycobilins are also used as food and cosmetic colorants due to their high yield [118]. 

Lutein is one of the most important carotenoids. Moreover, it is essential to the macula lutea in the retina and lens of eyes. Lutein industrial applications are as a colorant in food products [119]. Cancer, retinal degeneration, and cardiovascular diseases are some of the health applications [119]. As listed before the applications, the commercial potential of lutein from microalgae is high, but its large-scale production has not yet started to our knowledge [120]. Nonetheless, the basis for lutein production outdoors at a pilot scale for *Scenedesmus* has already been set up [121]. Carotenoids properties, which were discussed by Gille et al., [122], make them outstanding as functional foods. One of the most known is ß-carotene for its nature in sustaining growth and vision. Additionally, other carotenoids have been used as important food colorants [123]. That is the case of astaxanthin, another representative of the xanthophyll group of carotenoid pigments for its properties as a powerful antioxidant. It is important for humans to protect the skin from UV light as UV-induced photo-oxidation, antibody production, and anti-tumor therapy [124]. 

### 4.2. Nutraceutical Potentialities

Aztecs in Mexico used *Spirulina* sp. as food [125]. The application of these microalgae as food or dietary supplement has continued and have resulted in the research and finding of new species to be used in food applications. Besides direct consumption, derived products are used in food industry as colorants, antioxidants, and natural preservatives. The following paragraphs describe the most representative used species and respective applications. More recently, microalgae were incorporated into pasta, snack foods, candy bars or gums, and beverages as well [85,126]. Owing to their diverse chemical properties, they can act as a nutritional supplement or represent a source of natural food colorants [126]. The most relevant strains to commercial applications are *Chlorella*, *Spirulina*, *Scenedesmus,* and *Nostoc*. The polysaccharides from type β-1,3-glucan are known for its properties of immune-stimulation, reduction of free radicals and blood lipids, this substance is produced by *Chlorella* [127]. In addition, glucan has also benefited the immune system, reduce depression, protect the skin, and has anticancer properties [83,85,109,110,111,112]. Finally, phycobiliproteins and chlorophylls can be found as a food additive produced by *P. cruentum* [128].

As mentioned before, *Spirulina* has been used as a supplement in human nutrition. It is worth mentioning as it produces linolenic acid, which humans cannot synthesize [126], proteins, β-carotene, thiamine, riboflavin, vitamin B12. *Spirulina* also showed to be active against hyperlipidemia, hypertension, oxidative stress, arthritis, and serum glucose levels [129]. One attempt to introduce *Spirulina* into diet was by including it into cookies; in this study, the antioxidant properties were explored [130]. The most common microalgae strains used in the industry are *Chlorella* and *Spirulina* thanks to their high protein content and respective aminoacid profile, nutritive value, and standardized growth protocols with high biomass yield [131]. Although *Scenedesmus* was considered a pioneer as a food source, recently its application has been limited, and some extracts have been used in desserts, fruit puddings, and soups [83]. Besides other nutrients, *Scenedesmus* produces eicosapentaenoic acid (EPA), vitamins, and essential minerals [132].

### 4.3. Bioactive Compounds

Microalga have become a research target since they are a rich source of bioactive compounds [133,134,135]. The activities of the secondary metabolites isolated from microalga include antibacterial, antifungal, antialgal, antiprotozoal, and antiviral (Table 3). For example, the cyanobacterium *Phormidium* sp. has been reported to inhibit the growth of different Gram-positive and Gram-negative bacterial strains, yeasts, and fungi [136]. *Lyngbya majuscula* [70] that produces polyketides, lipopeptides, cyclic peptides, and many others compounds that have activities such as anti-HIV, anticancer, antifeedant, antifungal, anti-inflammatory, antimicrobial, antiviral, etc. [71]. Other biological activities of these compounds include protein kinase C activators and tumor promoters, inhibitors of microtubulin assembly, antimicrobial and antifungal, and sodium-channel blockers [71]. Antifungal compounds include fisherellin A, hapalindole, carazostatin, phytoalexin, tolytoxin, scytophycin, toyocamycin, tjipanazole, nostocyclamide and nostodione produced by cyanobacteria belonging to *Nostocales* and *Oscillatoriales* (Table 3) [75].

It is also important to mention the production of long-chain polyunsaturated fatty acids (PUFAs) such as eicosapentaenoic acid (EPA), docosahexaenoic acid (DHA), and arachidonic acid (AA) are important for human diet [15]. Besides, PUFAs have been used to prevent and treat chronic inflammations diseases (e.g., rheumatism, skin diseases, and inflammation of the mucosa of the gastrointestinal tract) [70,71]. In addition, studies show a positive effect on cardio-circulatory diseases, coronary heart diseases, atherosclerosis, hypertension, cholesterol, and cancer treatment [83,137]. Arachidonic acid (ARA), an essential component of membrane phospholipids with a function of vasodilator, shows anti-inflammatory effects [138]. Moreover, ARA is necessary for the repair and growth of skeletal muscle tissue and makes it a powerful dietary component in support of the anabolic muscle formulations [139]. The inclusion of *Spirulina* in malnourished children has shown improvement against anemia by increasing hemoglobin, protein, and vitamin levels [140]. Additionally, phycocyanin, γ-linolenic acid, vitamins, phenolic compounds, and minerals can help with malnourished children [141]. The consumption of *S. platensis* and *S. maxima* showed an increase of lactic acid bacteria increase in the gastrointestinal tract [83].

### 4.4. Bioremediation Potentialities

The ability to metabolize or bio-transform chemicals is one of the many properties of microalgae. Some of the remarkable studies are shown in the following paragraphs, including treatment against petroleum, herbicides, wastewater, etc. The potential of using the microalgae as a tool and profit from it is huge. Cyanobacteria have shown great potential against surfactants and herbicides as well [142,143,144]. Radwan et al. [145] showed the degradation of petroleum by using a functionalized mat with microalgae, and fluometuron and lindane degradation were investigated by the group of Mansy and El-Bestway [146], showing promising results when using a wide variety of microalgae.

Wastewater treatment by microalgae for the reduction of different contaminants is another bioremediation potential. The case to reduce calcium and chloride by the use of *Oscillatoria* sp. was studied by Uma and Subramanian [147]. Nitrogen and phosphorous reduction in wastewater by microalgae production is a strategy by combining it with bioremediation of amino acids, enzyme, or food industry effluents. *Chlorella*, *Spirulina,* and *Scenedesmus* are some of the species most used in these systems to reduce the eutrophication in water bodies [148]. The strategy to use the exopolysaccharides (EPS) produced in high amounts by cyanobacteria and microalgae as emulsifiers are driving the researcher’s attention. It can be applied to oil, metal, and dye recovery. Further, Matsunaga et al., [149] used *Anabaena* sp. to remove dyes from textile effluent, and *Phormidium autumnale* was used to degrade indigo dye in 20 days [150].

In addition, exopolysaccharides produced by some cyanobacteria have the capacity of capturing heavy metals suspended in water. The proper function of the EPS needs high purity, which is achieved by ionic resins treatment [151]. Moreover, novel studies suggest green extractions as alternative methods to get high purity EPS using membranes, ultrasound, and microwave. The bio-adsorption process occurs when negative charges present in EPS interact with heavy metal ions producing bonds. Chelation of positively charged ions on the microalgal polysaccharides layer is due to a high crosslink number that promotes fast kinetics. In recent literature, it has been demonstrated that the chelation of zinc, copper, cadmium, lead, arsenic, chromium, and mercury for the potential applications for heavy metals removal currently known as biosorption [152,153].

### 4.5. Bio-Fuels

The use of microalgae to generate biofuels has huge potential due to its oil content, biomass, and hydrocarbons production. The use of microalgae to produce energy is wide, and the biological conversion method of fermentation to generate hydrogen, ethanol, biodiesel, and biogas are the most important [154]. Hydrogen is the most efficient and cleanest energy carrier and *Chlamydomonas*, *Arthrospira*, and *Chlorella* microalgae species possess all the characteristics to photo produce hydrogen gas (Khetkorn et al.) [155]. The increment of photobiological production of hydrogen is related to the carbon content in biomass [156,157,158]. Other strains of interest are *Anabaena* able to produce hydrogen [159]. Additionally, *S. platensis* can produce hydrogen in dark conditions with photobiological hydrogen production [157].

#### 4.5.1. Photosynthetically Production of Hydrogen

Cyanobacteria can produce hydrogen in two different ways. First, grown under nitrogen limiting conditions, as a byproduct of nitrogen fixation when the species nitrogenase-containing heterocysts. Second, a reversible activity of hydrogenases enzymes [160]. The two microalgae species studied for hydrogen production were *Anabaena* and *Scenedesmus*. When *Anabaena* was placed into a glass jar it produced hydrogen gas, and after a period of dark anaerobic “adaptation”, *Scenedesmus* sp. produces hydrogen at low rates, greatly stimulated by short periods of light [161,162].

#### 4.5.2. Biodiesel/Bioethanol

The substitute for conventional diesel is biodiesel, the result of transesterification of lipids. The production of biodiesel is currently done by processing oily seeds from palm, castor bean, sunflower, corn, and cotton, among others [163,164,165]. As discussed before, microalgae are a huge producer of fatty acids that can be converted into biodiesel [59]. For instance, around 50% of *Chlorella, Nannochloropsis, Dunaliella, Scenedesmus,* and *Scenedesmus* composition are lipids. Microalgae biofuel has a high calorific value, low viscosity, and low-density properties turning it in a more suitable biofuel than lignocellulosic materials [166].

Among other benefits, the use of ethanol as combustible reduces levels of lead, sulfur, carbon monoxide, and particulates [167]. Normally, ethanol is produced from sugar from byproducts of sugar cane and corn through fermentation of biomass [168]. Microalgae have the fermentable potential substrate since they have high levels of carbon compounds, directly available or after a pre-treatment. Some fermentable microalgae, such as *Chlorella* sp., *Oscillatoria* sp., and *S. platensis,* have already been used to produce ethanol [169]. The ethanol should then be purified and used as efficient fuel and the CO_2_ can be recycled in the cultivation of more microalgae or use of residual biomass in the process of anaerobic digestion [170]. 

### 4.6. Antioxidants

Microalgae are rich in vitamins [85,112]. They can also accumulate vitamin E and fat-soluble phenols with antioxidant properties. Vitamin E has a wide range of applications. Some of its applications in medicine are to treat cancer, heart, eye, Alzheimer’s, Parkinson’s disease, and other medical conditions [171]. Harvested microalgae are used in the food industry as added preservatives, health-improving additives, and for photoprotection in skin creams [172]. 

Phycocyanin purified from cyanobacterium *Synechococcus* sp. R42DM showed antioxidant activity in vitro and in vivo. The cyanobacterium was isolated from a polluted industrial site in India [141]. The conditions showed stability in thermal and oxidative stress with *Caenorhabditis elegans* [141]. *Geitlerinema* sp. H8DM is another microalgae that produced a variation of phycocyanin [173].

### 4.7. Phycotoxins

One of the groups of microalgae responsible for producing phycotoxins are dinoflagellates that lead to harmful algal blooms and “red tides” [174]. Although, the same microalgae can produce a wide spectrum of secondary metabolites that may be applied to therapy as antitumor, antibiotic, antifungal, immunosuppressant, cytotoxic, and neurotoxic named as phycotoxins [175]. The other group is cyanobacteria. For example, *Nostoc* species are responsible for freshwater toxins with a potential pharmaceutical use such as borophycin used against human carcinoma, borophycin-8 as antibiotic, apratoxin A as a cytotoxin and anticancer, among another more than 30 metabolites [176,177]. Further, *Anabaena* produces bromoana-indolone, an antibiotic compound, along with balticidins and laxaphycins, antifungal metabolites against *Candida albicans*, *Penicillium notatum*, *Saccharomyces cerevisiae*, and *Trichophyton mentagrophytes*. The same species *Anabaena* produces sulfoglycolipids, antivirals that can inhibit the HIV-1 virus. Other antivirus families are lectins, e.g., cyanovirin-N which are produced by *Nostoc* sp. that prevents HI virus infections. It is effective against influenza A and B as well [178].

## 5. Opportunities for Improvement

Drug discovery through microalgae biotechnology is under-represented in the current pharmaceutical industry. Nevertheless, drug development by natural means like microalgae gives several advantages such as water solubility, membrane permeability, biodegradability, bioavailability, and biocompatibility [7]. Aquaculture feeds require a large volume of biomass for fish, mollusks, and crustaceans. However, accumulation of biomass can be difficult and expensive, but the inclusion of microalgal increases dietary value to feed with essential amino acids, fatty acids, high-quality protein, vitamins, micronutrients, and carotenoids [179]. Products from fisheries and aquaculture combined are supplying the world with 142 million tons of protein every year with a market value of 106 billion dollars calculated in 2008 [179].

Biofuels in the form of gas and liquid products are gaining impact by the world regulations of green economies. The use of microalgae to produce enzymes to be included in specific catalyze processes to generate combustibles. For instance, biodiesel is an opportunity with enzyme-chemistry [180]. More important is to address high-value products, as turbocine with more caloric power for future perspectives and include secondary products generated in the process to stack value and impact the current market. Carbon dioxide capture is another opportunity for microalgae. The utilization of microalgae cultures in industrial processes can capture harmful gas emissions [181]. The accumulation of carbon dioxide can be applied under specific conditions to match with proper algal strains in their mechanisms of photosynthesis, leading to a decrease in pollution and produce biomass [182]. 

The biopolymer field is a branch of chemistry and material science where the application of novel technologies are required to produce bioplastics. An urgent large-scale production of biodegradable materials for common use, like packaging and containers, is far from current technology. However, the application of biocompatible and biodegradable plastics with zero toxicity to mammal cells offers the initial impulse to study the production of chitosan, cellulose, polyhydroxyalkanoates, and other biopolymers by microalgae. A hot topic is the utilization of scaffolds to support cellular growth in prosthesis and patches to treat skin burns, and missing or destroyed bones [183]. Biosorption is a removal process of potentially toxic elements where adsorption, chelation, ion exchange, and surface precipitation may occur. Through microalgae, heavy metals can be removed from municipal and industrial wastewaters [153]. Its potential increases with the lack of fresh water and recently with the detection of emerging contaminants as pesticides and drug wastes from pharmacological industries [184]. 

## 6. Concluding Remarks and Future Perspectives

Discovery, isolation, and preservation of novel microalgae strains are evident as we showed in Table 1, where a large number of species were enumerated in recent years. Still, many more are waiting to be discovered in the vast fresh and marine water resources in Mexico. An opportunity to generate green solutions to local problems through science, innovation, technology development, and transfer becomes one of the most important objectives for the field of microalgae biotechnology applications. Investment in biodiversity has a relevant impact on preserving and studying the natural resources in the country. However, attractive applications for industry investment are imperative to have relevant participation in research. In addition, the involvement of industry, society, and the research community helps to protect Mexico’s biodiversity. A green economy means political strategies towards a low-carbon economy, resource efficiency, green investments, technological innovation and more recycling, green jobs, poverty eradication, and social inclusion. The whole idea points to a higher support for novel technologies that help in the mentioned point, especially towards the knowledge development of involving local ecosystems. Local and global problems list include waste management, water treatment and emerging pollutants with potential opportunities to provide innovative solutions. A close collaboration between society, industry, and research institutions will lead to the path for a sustainable development.

## Figures and Tables

**Table 1:** Microalgae biodiversity in Mexico.

State	Municipality/Location	Microalgae	References
Baja California	Ensenada	*Aphanocapsa marina*	[19]
		*Komvophoron* sp.	
		*Phormidium* sp.	
		*Tetraselmis suecica*	
		*Heterococcus* sp.	
		*Amphora* sp. (7)	
		*Cymbella* sp. (2)	
		*Navicula* sp. (4)	
		*Diploneis* sp.	
		*Grammatophora angulosa*	
		*Synedra* sp.	
Veracruz	Catemaco	*Aphanothece comasii*	[17]
		*Cyanotetras aerotopa*	
		*Cylindrospermopsis catemaco*	
		*Cylindrospermopsis taverae*	
		*Planktolyngbya regularis*	
San Luis Potosí		*Cyanobacterium lineatum*	
Puebla	Alchichica	*Cyclotella alchichicana*	
		*Chroococcus deltoids*	
Baja California, Colima, Michoacan, Guerrero, Tamaulipas, Veracruz, Hidalgo, Mexico city	Ensenada, Manzanillo, Lazaro Cardenas, Acapulco and Zihuatanejo, Laguna de Carpintero, Garrapatas and Barberena estuaries, Catemaco and Chalchoapan Lakes, Vicente Aguirre dam, Xochimilco Lake	*Alexandrium tamarense* *Amphidinium* sp*.* *Cochlodinium polykrikoides* *Heterocapsa pigmea* *Gyrodinium instriatum* *Gymnodinium catenatum* *Karlodinium veneficum* *Prorocentrum gracile* *Prorocentrum micans* *Prorocentrum triestimum* *Prorocentrum mexicanum* *Prorocentrum rathymum* *Protoceratium reticulatum* *Scrippsiella trochoidea* *Bacillaria paxilifera* *Cylindrotheca closterium* *Pseudonitszchia delicatisima* *Chattonella marina*	[21]
Mexico City	Mexico City	*Spirulina maxima*	[22]
Baja California Sur	La Paz	*Rhabdonema* sp.	[23]
		*Schizochytrium* sp.	
		*Nitzchia* sp.	
		*Navicula* sp.	
		*Grammatophora* sp.	
Mexico City	Mexico City	*Spirulina platensis*	[24]
		*Spirulina maxima*	
Queretaro	Not specified	*Oscillatoria* sp.	[25]
Guanajuato	Valle de Santiago	*Actinastrum* sp.	[26]
Baja California Sur	La Paz	*Lyngbya* sp.	[27]
		*Oscillatoria* sp.	
		*Microcoleus* sp.	
		*Anabaena* sp.	
Nuevo León	Apodaca	*Scenedesmus* sp.	[28]
	Cadereyta	*Chlorella sorokiniana*	
Campeche	El Carmen	*Anabaena* sp.	[29]
		*Oscillatoria* sp.	
		*Anabaena* sp.	
		*Cylindrospermopsis cuspis*	
Oaxaca	Zipolite	*Dermocarpella* sp.	[30]
Morelos	Tlaquiltenango	*Nostoc* sp.	[31]
Mexico City	Mexico City	*Desmodesmus* sp.	[32]
Coahuila	Cuatrociénegas	*Scenedesmus* sp.	[33]
Mexico City	Mexico City	*Microcystis*	[34]
Michoacan	Michoacan	*Codium giraffa*	[35]
Guerrero	Papanoa	*Codium giraffa*	[36]
Michoacán	Los Azufres	*Trebouxiophyceae* sp.	[37]

**Table 2:** Compounds from microalgae extracted by novel green techniques.

Compound(s) of Interest	Species	Extraction Technique	References
C-phycocyanin Pigments	*Spirulina maxima*	Ultrasound	[39]
β-carotene	*Chlorella* sp.	Ultrasound	[40]
Polyphenols Flavonoids	*Spirulina platensis*	Microwave and Ultrasound	[41]
Lipids	*Scenedesmus* sp.	Microwave	[42]
Lipids	*Scenedesmus obliquus &* *Scenedesmus obtusiusculus*	Supercritical-CO_2_	[43]
Oil	*Spirulina platensis*	Supercritical-CO_2_	[44]
Docosahexaenoic acid	*Schizochytrium limacinum*	Supercritical-CO_2_ -vegetable oil	[45]
Lipids, Carotenoids	*Chlorella vulgaris*	Supercritical-CO_2_	[46]
Lipids	*Chlorella vulgaris*	Ultrasound & Bligh and Dyer method	[47]
β-carotene	*Spirulina platensis*	Ultrasound	[48]
Vitamins Phycocyanin Fatty Acids	*Spirulina platensis*	Microwave	[49]
Lipids	*Chlorella* sp.	Microwave and Ultrasound	[50]
Long-chain PUFAs	*Schizochytrium* sp.	Supercritical-CO_2_	[51]
Carotenoids Fatty Acids	*Spirulina platensis*	Microwave and Supercritical-CO_2_ -etOH	[49]
C-phycocyanin	*Spirulina platensis*	Ultrasound	[52]
Neutral Lipids	*Chlorella vulgaris &* *Nannochloropsis oculata*	Supercritical-CO_2_	[53]
Chlorophyll	*Chlorella vulgaris*	Ultrasound	[54]
Lipids	*Scenedesmus obliquus*	Ultrasound + solvent	[55]

**Table 3:** Summary of existing high-value products from Mexican microalgae species.

Microalgae	Bioactive Compounds	Biological Activity	References
*Oscillatoriaceae* sp.	Malyngolide	Antibacterial	[70]
	Lyngbyatoxins	PKC activator	
	Debromoaplysiatoxin	Inflammatory	
*Lyngbya majuscula*	Curacin A	Microtubulin assembly inhibitors	[71]
	Kalkitoxin	Sodium channel blocker	
	Cyclic polypeptide	Anti-HIV activity	
*Oscillatoria raoi*	Acetylated sulfoglycolipids	Antiviral	[72]
*Spirulina platensis*	Spirulan	Antiviral	[73]
*Nostocaceae* sp.	Nostocyclamide	Antifungal	[74]
*Chroococcaceae* sp.	Kawaguchipeptin B	Antibacterial	[75]
*Mycrosistis aeuregonosa*			
*Scenedesmus* sp.	Lutein	Anti-oxidant	[76,77,78,79,80]
*Spirulina (Arthrospira)*	γ-Linolenic acid (GLA)	The integrity of tissues, delay of aging	[81]
*Spirulina (Arthrospira)*	Phycocyanin	Antioxidant, anti-inflammatory	[15,82]
*Tetracelmis suecica*	α- tocopherol	Antioxidant	[15]
*Chlorella* sp.	Galactose, rhamnose, mannose, arabinose, N-acetyl glucosamide and N-acetyl galactosamine	Immune stimulatory activity	[83]
*Spirulina platensis and Anabaena* sp.	Proteins		[84,85,86]
*Anabaena* sp.	Superoxide dismutase (SOD)	Antioxidant, anti-inflammatory	[87,88,89,90]
*Spirulina* sp.	Vitamin C; vitamin K; vitamins $B_{12}$, A and E; α-tocopherol	Antioxidant; blood cell formation; blood clotting mechanism	[15,91]
*Chlorella* sp.	Lutein, zeaxanthin, canthaxanthin	Antioxidant	[15,92]
*Lyngbya majuscula*	Microlin- A	Immunosuppressive	[93]
*Chlorella sorokiniana* and *Scenedesmus* spp.	Mycosporine-like amino acids (MAA)	UV-screening agent; sunscreen	[94,95,96,97]
*Chlorella* sp.	α-carotene Astaxanthin	Lower risk of premature death	[98]
*C. sorokiniana*	β-carotene	Food colorant; antioxidant property; cancer preventive properties; prevent night blindness; prevent liver fibrosis	[99,100]
*Tretraselmis* spp.	Zeaxanthin	Protect eye cells; antioxidant activity; neutralizing the free radicals	[101,102]
*Nitzschia* spp.	Triglycerides and hydrocarbons	Biofuels	[95,103,104]
*Tetraselmis* spp. and *T. suecica*	Arachidonic acid (AA) Eicosapentaenoic acid (EPA)	Nutritional supplements, aquaculture feeds	[105,106]
*T. suecica*	Sterols	Antidiabetic; anticancer; anti- inflammatory; anti-photoaging; anti-obesity; anti-inflammatory; antioxidant activities	[107,108]
*Chlorella* spp. and *C. sorokiniana*	Vitamin B Vitamin C	Decrease fatigue; reducing depression; protect against heart disease; protect the skin; anticancer activity Protect against cardiovascular disease; prenatal health problems; prevent from the eye disease; protect against skin wrinkling	[85,99,109,110,111,112]
*C. sorokiniana* and *T. suecica*	Vitamin E	Protect against toxic pollutants; Premenstrual syndrome protects against eye disorders; anti- Alzheimer’s disease; anti- diabetic properties	[85,98,111,113]
